# Methylation level of potato gene *OMT30376* regulates tuber anthocyanin transformations

**DOI:** 10.3389/fpls.2022.1021617

**Published:** 2022-10-07

**Authors:** Huiling Zhang, Yanan Zhao, Xijuan Zhao, Zhonghua Zhang, Ju Liu, Minghui Shi, Botao Song

**Affiliations:** ^1^ College of Horticulture and Plant Protection, Henan University of Science and Technology, Luoyang, China; ^2^ Key Laboratory of Horticultural Plant Biology, Ministry of Education, Huazhong Agricultural University, Wuhan, China; ^3^ Key Laboratory of Potato Biology and Biotechnology, Ministry of Agriculture and Rural Affairs, Huazhong Agricultural University, Wuhan, China; ^4^ Yichang Agricultural Technology Extension Center, Yichang, China

**Keywords:** *OMT30376* gene, methylation level, gene expression, anthocyanin transformation, potato

## Abstract

After anthocyanin synthesis, a variety of anthocyanin compounds are produced through further methylation, glycosylation, and acylation. However, the effect of the potato methylase gene on anthocyanin biosynthesis has not been reported. Red and purple mutation types appear in tubers of the potato cultivar ‘Purple Viking’ with chimeric skin phenotypes. In this study, transcriptome and anthocyanin metabolome analyses were performed on skin of Purple Viking tubers and associated mutants. According to the metabolome analysis, the transformation of delphinidin into malvidin-3-O-glucoside and petunidin 3-O-glucoside and that of cyanidin into rosinidin O-hexoside and peonidin-3-O-glucoside were hindered in red tubers. Expression of methyltransferase gene *OMT30376* was significantly lower in red tubers than in purple ones, whereas the methylation level of *OMT30376* was significantly higher in red tubers. In addition, red skin appeared in tubers from purple tuber plants treated with S-adenosylmethionine (SAM), indicating the difference between purple and red was caused by the methylation degree of the gene *OMT30376*. Thus, the results of the study suggest that the *OMT30376* gene is involved in the transformation of anthocyanins in potato tubers. The results also provide an important reference to reveal the regulatory mechanisms of anthocyanin biosynthesis and transformation.

## Introduction

Potato (*Solanum tuberosum* L.) is the fourth largest crop after wheat, rice, and corn and is used as a grain, vegetable, feed, and industrial raw material. Pigmented potato is rich in anthocyanins, which give tuber skin and flesh color, including purple, blue, and red (Fossen and Andersen, 2000). Biosynthesis and accumulation of anthocyanins can increase plant resistance to different biological and abiotic stresses, such as low temperature, ultraviolet radiation, drought stress, and diseases (Liu et al., 2019). Anthocyanins also benefit human health because of strong antioxidant capacity (Heim et al., 2002; Khoo et al., 2017). The antioxidant capacity of pigmented potato tubers is three to five times that of yellow and white tubers (Brown, 2005). Consuming pigmented potatoes can also significantly reduce the incidence rate of prostate cancer and colon cancer (Reddivari et al., 2007).

Anthocyanins are synthesized through the flavonoid pathway, which includes both structural and regulatory genes. Structural genes encode enzymes that primarily catalyze the formation of anthocyanin through three main synthetic pathways, namely cyanidin, pelargonidin and delphinidin pathways (Liu et al., 2018). Further modification of anthocyanins, including methylation, glycosylation, and acylation of the three aglycones, can produce a variety of anthocyanin compounds and enhance color phenotypes. Those processes are catalyzed by anthocyanin O-methyltransferase (OMT), flavonoid 3-glucosyl transferase (3GT), and acyltransferase (AT), respectively (Rausher, 2008; Wessinger and Rausher, 2012). The tissue-specific expression of structural genes is mainly regulated by the transcription factors R2R3-MYB, basic helix-loop-helix (bHLH), WD-repeats (WDR), and the MYB-bHLH-WDR (MBW) complex (Hichri et al., 2011).

Gene methylation is the process of transferring an activated methyl from S-adenosylmethionine (SAM) to N-, C-, O- or s-nucleophiles of receptor molecules, which occur widely in plants and animals (Klimasauskas and Weinhold, 2007). Methylation is an epigenetic modification that can affect many biological processes, including growth and development of fruits (Ma et al., 2018; Guo et al., 2019) and anthocyanin biosynthesis (Jiang et al., 2020). The anthocyanin content of a yellow-skinned apple mutant was lower than that of its parent because of the higher methylation level of the *MdMYB10* promoter (El-Sharkawy et al., 2015). Similar results are also observed in a red-bud apple (Xu et al., 2012). When the key regulatory gene in anthocyanin biosynthesis in red-fleshed radish, *RsMYB1*, is mutated because of a hypermethylated transposon sequence in the promoter, white-fleshed radish is produced. Methylation of the *RsMYB1* promoter leads to a decrease in gene expression and affects the biosynthesis and accumulation of anthocyanin (Wang et al., 2020).

Most methyltransferases act on hydroxyl and carboxyl groups and are called O- methyltransferases (OMTs). The OMTs can catalyze specific substrates to produce a wide variety of anthocyanin compounds that enrich color phenotypes (Wessinger and Rausher, 2012). Several OMT genes involved in the formation of O-methylated anthocyanins have been previously reported (Sakata et al., 1995; Hugueney et al., 2009). Genetic analysis of anthocyanin biosynthesis in petunia shows that *MT1/MT2* and *MF1/MF2*, two pairs of repetitive genes, are responsible for the methylation of anthocyanin molecules (Wiering and Devlaming, 1977). Owing to the action of OMTs, the content of anthocyanin components containing methoxyl and hydroxyl groups changes. In studies on tree peony petals, as the number of methoxyl groups increases, the transformation from red to purple also increases (Sakata et al., 1995). The red–purple-flowered cyclamen mutant appeared in purple-flowered cyclamen following ion-beam irradiation (Kondo et al., 2009), and the major anthocyanins in the petals of red–purple-flowers and purple-flowers were delphinidin 3, 5-diglucoside and malvidin 3, 5-diglucoside, respectively. In further research, *CkmOMT2* catalysis of the 3′ or 3′5′ O-methylation of the B-ring of anthocyanin substrates was only detected in purple-flowered cyclamen, and not in the red–purple-flowered mutant. Therefore, defective *CkmOMT2* gene expression was responsible for the change in anthocyanin composition and flower coloration in mutants (Kondo et al., 2009). Genes of O-methyltransferases are also found in purple-flowered (*PsAOMT*) and red-flowered (*PtAMOT*) paeonia plants. Owing to the difference of a single amino acid between PsAOMT and PtAMOT, there are significant differences in gene expression and enzyme activity, which ultimately affect the formation of purple (Du et al., 2015). Thus, previous results show that OMT genes have important roles in the transformation from red anthocyanin to purple anthocyanin. However, whether potato OMTs regulate tuber anthocyanin transformation remains unclear.

In the present study, the transcriptome and anthocyanin metabolic components of the potato cultivar Purple Viking and its red and purple mutant tubers were analyzed. The results showed that methylation level of the O*MT30376* gene might affect anthocyanin transformation in tubers. The study will provide a theoretical basis to reveal the molecular mechanism regulating anthocyanin biosynthesis in potato tubers.

## Materials and methods

### Plant materials

The potato (*Solanum tuberosum* L.) ‘Purple Viking’ and its mutants were grown in 24-cm-diameter plastic pots in a greenhouse with 16 h of light per day supplied by lamps. Three biological replicates were made for each material for subsequent anthocyanin content and transcriptome analysis. At 80 d after planting, tubers were harvested according to skin color. Skin (1 mm thick) of Purple Viking tubers (PurS), flesh of Purple Viking tubers (PurF), Skin (1 mm thick) of red mutant (M-R) and purple mutant (M-P) of Purple Viking were sampled respectively, and frozen immediately in liquid nitrogen and stored at -80°C until use.

### Anthocyanin extraction and metabolomics analysis

Powder, 100mg, was extracted with 1.0 mL 70% aqueous methanol overnight at 4°C. Extracts were centrifuged at 10,000 ×g for 10 min and then filtrated (0.22-μm pore size). Samples were analyzed using an LC-ESI-MS/MS system (HPLC, Shim-pack UFLC SHIMADZU CBM30A system). The LC-ESI-MS/MS analysis was performed by Wuhan MetWare Biotechnology Co., Ltd., (www.mettware.cn). Metabolite content data were normalized by the range method, and accumulation patterns of metabolites among different samples were analyzed by the R package. Fold Change ≥ 2 and ≤ 0.5 were set as the threshold for significantly differential metabolites.

### RNA isolation and library construction

Total RNA was isolated using a PLANTpure Universal RNA Kit (Aidlab, Beijing, China) according to the manufacturer’s instructions, and then, post-treatment with a DNase Digestion Kit (Aidlab, Beijing, China) was used to remove genomic DNA contaminants. The mRNA was purified from total RNA with poly-T oligo-attached magnetic beads. First-strand cDNA was synthesized under elevated temperature in NEBNext first strand synthesis reaction buffer (5×) using random hexamer primers and M-MuLV Reverse Transcriptase. Second-strand cDNA synthesis was subsequently performed using RNase H and DNA Polymerase I. Library fragments were purified with an AMPure XP system (Beckman Coulter, Beverly, USA). Then, PCR was performed with Phusion High-Fidelity DNA polymerase, Universal PCR primers, and Index (X) Primer. The PCR products were purified (AMPure XP system), and library quality was assessed on an Agilent Bioanalyzer 2100 system. Library products were then sequenced *via* an Illumina HiSeqTM 2000 (San Diego, CA, USA).

### Sequence data filtering, *de novo* assembly, and annotation

Clean reads were obtained by removing adapters, and reads containing ploy-N and those of low-quality from raw reads. Simultaneously, Q20, Q30, GC content, and sequence duplication level of clean reads were calculated. Clean reads were then mapped to the potato DM reference genome (v4.03, http://spuddb.uga.edu/pgsc_download.shtml) with HISAT2 (v2.1.0, Kim et al., 2015). Gene function was annotated based on public databases, including the National Center for Biotechnology Information (NCBI) non-redundant protein sequences (NR) and Swiss-Prot (Apweiler et al., 2004), Protein family (Pfam) (Finn et al., 2014), Clusters of Orthologous Groups of proteins (COG) (Tatusov et al., 2000), eukaryotic Orthologous Groups of proteins (KOG) (Koonin et al., 2004), and Kyoto Encyclopedia of Genes and Genomes (KEGG) (Kanehisa et al., 2004) databases.

### Differentially expressed gene analyses

Fragments Per Kilobase of transcript per Million fragments mapped (FPKM) were used to estimate gene expression level. Differential gene expression among different samples was estimated by Cuffdiff (v2.0.0). Thresholds for significantly differential expression were false discovery rate (FDR) ≤ 0.05 and fold change ≥2. The KEGG pathways enrichment analysis of differentially expressed genes (DEGs) was performed by KOBAS software (Mao et al., 2005).

### Reverse-transcription quantitative PCR

Isolation of RNA, reverse transcription, and reverse-transcription quantitative PCR (RT-qPCR) were conducted according to a previously method (Zhang et al., 2020). Primers were designed using the NCBI Primer-BLAST (https://www.ncbi.nlm.nih.gov/tools/primer-blast/) and are listed in **Table 1**. The RT-qPCR was performed on a CFX96™ real-time PCR system (Bio-Rad, USA) using a TransStart Top Green qPCR SuperMix kit (Transgen, Beijing, China). The potato gene Ef1α (GenBank: AB061263) was selected as the control. The relative expression of individual gene was calculated with the 2^−ΔΔCT^ method (Livak and Schmittgen, 2001).

**Table 1:** The primer sequences for PCR and RT-qPCR.

Primer name	Primer sequence (5′-3′)	Genbank ID
OMT10040-F	GCGCTTAACCTAACCCGAAA	PGSC0003DMG400003935^A^
OMT10040-R	TGTGGTGCAGTCAGAGTCAT	
OMT30376-F	ACGTTCAATGCGTTTGCTTC	PGSC0003DMG400011624 ^A^
OMT30376-R	AGAGGTAAGGGGAACAAAGTGT	
AT26230-F	CTAACCCACCAGAAGGCTCA	PGSC0003DMG400010117 ^A^
AT26230-R	ACCGTGTATTCCTCCGGTTT	
AT69755-F	TCACCGGAAACAATGGATGC	PGSC0003DMG400027127 ^A^
AT69755-R	AAAATCACAAAGAGCAAAAGCTACT	
OMT44353-F	TGAAAGACACTGCCCATTGC	PGSC0003DMG400017221 ^A^
OMT44353-R	TCGCCTTCCAAACCTCATCT	
FLS-F	ACACCGCGTCACTTTCCTAT	PGSC0003DMG400014093 ^A^
FLS-R	TCAAGGCTGTTGTTGCACTC	
bHLH96-F	GAACAGCAGGCACAATTGGA	PGSC0003DMG400028578 ^A^
bHLH96-R	TTCGTTCAACGGCAATGTGT	
nsLTP1-F	TGTTGCGGTGGAGTTAGGAA	PGSC0003DMG400001904 ^A^
nsLTP1-R	GACCAGCAGCTTTGCTGTAA	
DMT03581-F	TAATTGCGCACTTTGTGCCT	PGSC0003DMG400001415 ^A^
DMT03581-R	GGAGGTGATCCAGGTCAAGA	
Ef1α-F	ATTGGAAACGGATATGCTCCA	AB061263^B^
Ef1α-R	TCCTTACCTGAACGCCTGTCA	
OMT10040M-F	CCACTCCATCACTCATTGTAAGCC	
OMT10040M-R	CGATATCTGAATAAGTTCTGATGAATGTG	
AT26230M-F	TACATTGCTATTTTGGTTTCATCGT	
AT26230M-R	AAGAGAAACAGAAACATACAACATGATAA	
OMT30376M-F	TTTTCTAAAGTCAAACCCAAATCCT	
OMT30376M-R	CGATGAGCTTAACTTCAAGCCAC	

^A^Gene from the Potato Genome Sequence Consortium database.

^B^Gene from the National Center of Biotechnology Information database.

### Methylation assay

Methylation and demethylation treatments were applied. Potato M-P and M-R genotypes were induced to form tubers in MS medium containing 8% sucrose under a short-day photoperiod (8 h day/16 h night). In the methylation treatment, 30-µM S-adenosylmethionine (SAM) was added to sterilized MS medium, whereas in the demethylation treatment, 40-µM azacitidine (5-azaC) was added according to previous study (Ai et al., 2021). Water was used as the control. Thirty plantlets were used for each treatment. After 6 to 8 weeks, chimeric color of red and purple tubers (MPS) appeared in M-P plantlets cultured in the medium containing SAM. Three MPS tubers were planted separately to form three independent lines, named MPS-1, MPS-2, and MPS-3, respectively.

To detect methylation level, genomic DNA was isolated from leaves of M-P and M-R, red skin of Purple Viking tuber (Pur-R), purple skin of Purple Viking tuber (Pur-P), red skin of MPS-1 (MPS-1-R) and MPS-2 (MPS-2-R) tuber, and purple skin of MPS-1 (MPS-1-P) and MPS-2 (MPS-2-P) tuber using a Plant Genomic DNA Extraction Kit (DP305; TIangen, Beijing, China) following the manufacturer’s instructions. The gDNA was digested by the restriction enzyme *Hpa*II which is sensitive to the methylation of gene sequences. Then, PCR amplification was conducted with primers (MT30376M-F/R) spanning GC island sequences using gDNA before and after enzyme digestion as a template. The methylation level of target genes was determined according to the presence or absence of the target band in PCR products.

## Results

### Anthocyanin transformation was different between Purple Viking and its mutants

**Figure 1:**
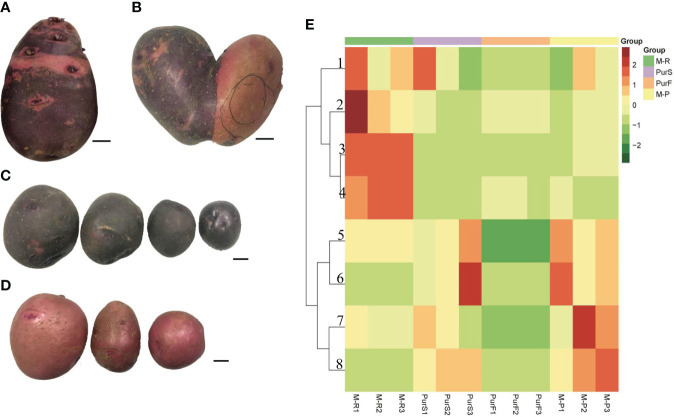
Tuber phenotype and anthocyanin content of potato cultivar Purple Viking and its mutants. **(A)** Phenotype of Purple Viking wild-type tuber. **(B)** Phenotype of Purple Viking chimeric tuber. **(C)** Tuber phenotype of Purple Viking red mutant (M-R). **(D)** Tuber phenotype of Purple Viking purple mutant (M-P). Bars=1cm. **(E)** Heat map of eight anthocyanins in tubers of Purple Viking and its mutants. 1: cyaniding; 2: delphinidin; 3: pelargonidin; 4: pelargonidin 3-O-beta-D-glucoside; 5: rosinidin-O-hexoside; 6: malvidin-3-O-glucoside; 7: rosinidin-O-hexoside; 8: petunidin 3-O-glucoside.

The tuber skin of Purple Viking was a chimeric color of red and purple (**Figure 1A**). A special tuber of Purple Viking with skin that was red on one side and purple on the other was obtained (**Figure 1B**), and the bud eyes of red and purple parts were planted separately. The tuber obtained by propagating buds from the purple part had pure purple skin (M-P) (**Figure 1C**), and the tuber obtained by propagating buds from the red part had pure red skin (M-R) (**Figure 1D**).

**Figure 2:**
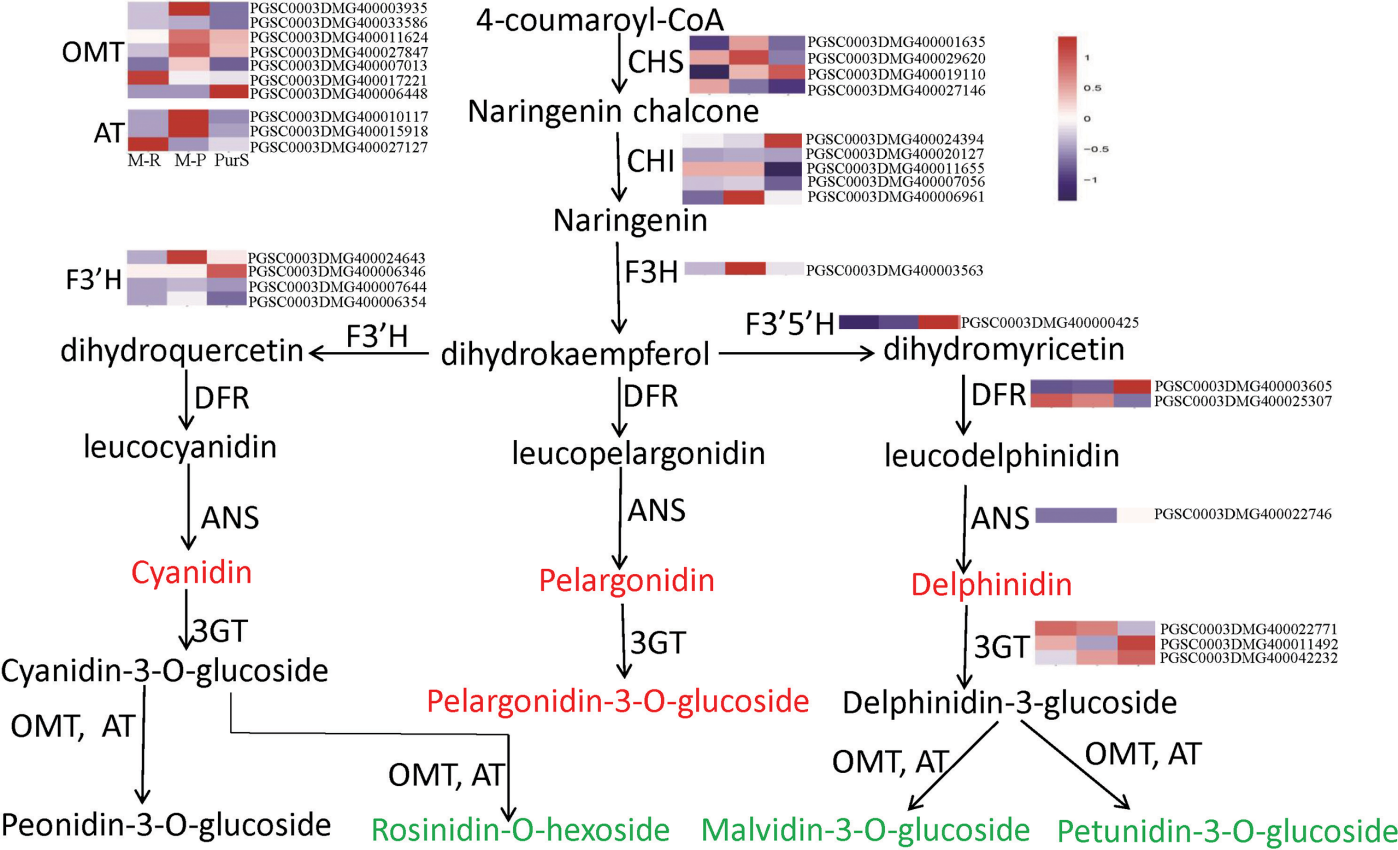
Metabolites and heat map of anthocyanin biosynthetic genes. Red font indicates that the content of anthocyanins in red tubers is higher than that in purple tubers, and green font indicates that the content of anthocyanins in red tubers is lower than that in purple tubers.

To determine differences in anthocyanin composition and content between purple and red tubers, anthocyanin metabolites of M-R and M-P were analyzed by HPLC. The skin and flesh of Purple Viking tubers (abbreviated as PurS and PurF, respectively) were used controls. Eight types of anthocyanins were detected, including rosinidin-O-hexoside, peonidin-3-O-glucoside, delphinidin, pelargonidin, malvidin-3-O-glucoside, petunidin 3-O-glucoside, pelargonidin 3-O-beta-D-glucoside, and cyaniding. Relative contents in each sample are shown in **Table S1**. According to cluster analysis of anthocyanin contents in each sample, contents of cyanidin, delphinidin, pelargonidin, and pelargonidin 3-O-beta-D-glucoside were higher in M-R than in M-P, whereas contents of the other four anthocyanins were higher in M-P (**Figure 1E**). According to the anthocyanin biosynthesis pathway, in M-R, the transformation of cyanidin into rosinidin O-hexoside and peonidin-3-O-glucoside and that of delphinidin into malvidin-3-O-glucoside and petunidin 3-O-glucoside were hindered (**Figure 2**).

### Differentially expressed genes in pure red skin and pure purple skin tubers

**Figure 3:**
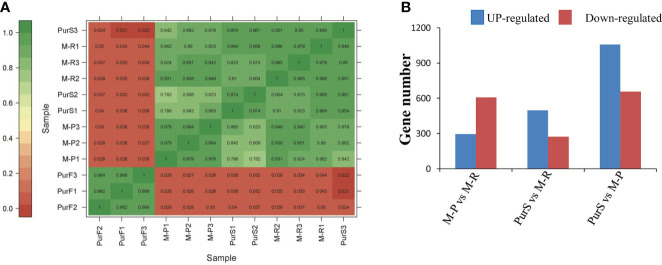
Overview of transcriptome analysis. **(A)** Correlation analysis of RNA-seq data of different tuber samples. **(B)** Number of differentially expressed genes (DEGs) in each comparison.

To further explore the molecular mechanisms that formed red and purple anthocyanins in tubers, the cDNA libraries of M-R, M-P, PurS, and PurF were constructed for transcriptomic analysis, with PurS and PurF used as controls. There was a strong correlation among biological repeats in the transcriptome, with Pearson’s correlation coefficient (*r^2^*) greater than 0.954 (**Figure 3A**). Number of clean reads per library ranged from 19,776,183 to 27,106,135, and 80.43% to 89.66% of reads mapped to the potato reference genome. At least 94.38% of reads in each library scored Q30 (0.02% error rate) or above, and the average GC content across all libraries was 43.00% (**Table S2**). The data demonstrated that reads were of high quality and were suitable for further differential gene expression analysis.

**Figure 4:**
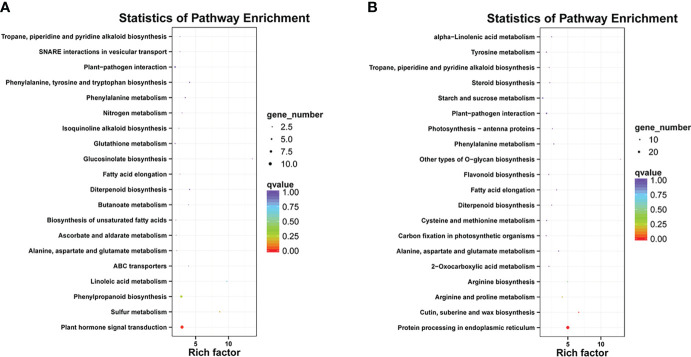
Kyoto Encyclopedia of Genes and Genomes (KEGG) enrichment analysis of **(A)** up-regulated and **(B)** down-regulated differentially expressed genes (DEGs) between red mutant (M-R) and purple mutant (M-P) tubers.

Differentially expressed genes in different libraries were analyzed to identify genes likely involved in the accumulation of different anthocyanins. Based on thresholds fold change ≥ 2 and FDR < 0.01, 296, 497, and 1,058 genes were up-regulated in the comparisons M-P vs M-R, PurS vs M-R, and PurS vs M-P, respectively, and 607, 273, and 657 genes were respectively significantly down-regulated (**Figure 3B**). The DEGs were identified in order to analyze the differential pathways and genes controlling the formation of red and purple anthocyanins in tubers. Thus, KEGG pathways enrichment analysis was performed to analyze up-regulated and down-regulated genes in the M-P vs M-R comparison. Thirty-nine enriched pathways were associated with the 296 up-regulated genes. The first 20 pathways of the enrichment analysis are shown in **Figure 4A**. The most significantly enriched pathways were those associated with plant hormone signal transduction, sulfur metabolism, phenylpropanoid biosynthesis, and linoleic acid metabolism. Forty-seven enriched pathways were associated with the 607 down-regulated genes. The first 20 pathways of the enrichment analysis are shown in **Figure 4B**. The most significantly enriched pathways were those associated with protein processing in endoplasmic reticulum; cutin, suberine, and wax biosynthesis; arginine and proline metabolism; and arginine biosynthesis.

According to expression patterns of anthocyanin synthesis structural genes in the transcriptomes, there was no significant difference in expression of most structural genes in M-R and M-P, including *CHI*, *F3’H*, *F3’5’H*, *DFR*, *ANS* and *3GT* (**Figure 2**). In further analysis, 236 genes had more than a 5-fold change in expression between M-R and M-P (**Table S3**), including the flavonol synthase gene (PGSC0003DMG400014093), glutathione s-transferase gene (PGSC0003DMG400015726), acyltransferase gene (PGSC0003DMG400010117), bHLH (PGSC0003DMG400028578), and WRKY (PGSC0003DMG400015104) transcription factors.

### Confirmation of differential gene expression by reverse-transcription quantitative PCR

**Figure 5:**
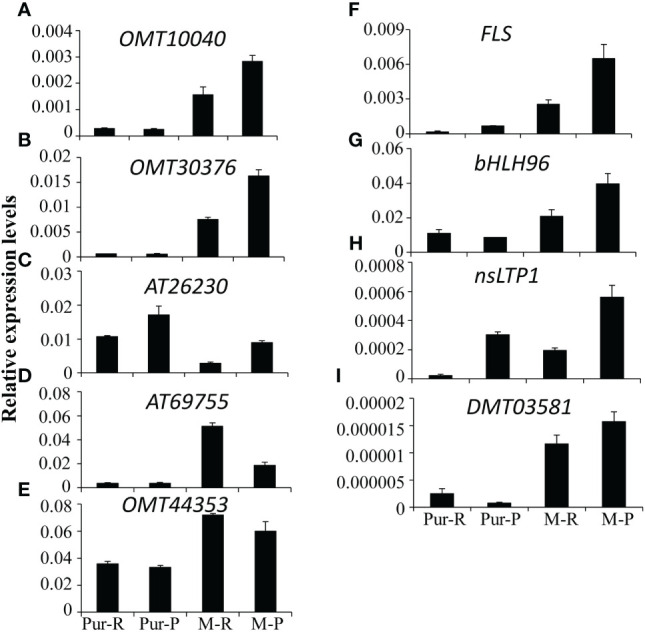
Quantitative analysis of transcript levels of differentially expressed genes (DEGs) in tubers skin of Purple Viking and its mutants. Each column represents the mean value ± standard error (*n* = 3).

According to differences in anthocyanin components between M-R and M-P, in the M-R tuber, the formation of rosinidin O-hexoside, malvidin-3-O-glucoside, and petunidin 3-O-glucoside was hindered. With consideration of the anthocyanin biosynthesis pathway (**Figure 2**), it was speculated that the limited formation of those compounds might be caused by functional differences in methyltransferase or acyltransferase. Of the DEGs in the transcriptome, seven genes encoded O-methyltransferase and three genes encoded acyltransferase. To verify the reliability of transcriptome data, expression patterns of the OMT and AT genes in different samples were detected by RT-qPCR. Four of the 236 genes with more than a 5-fold difference in expression between M-R and M-P were randomly selected for verification. The RT-qPCR results were consistent with the transcriptome results (**Figure 5**). Expression of two OMT genes (*OMT10040* (PGSC0003DMG400003935) and *OMT30376* (PGSC0003DMG400011624)) and one AT gene (*AT26230* (PGSC0003DMG400010117)) was significantly higher in M-P than in M-R (**Figures 5A–C**). It was speculated that their higher expression in M-P tubers might promote the rapid accumulation of rosinidin O-hexoside, malvidin-3-O-glucoside, and petunidin 3-O-glucoside.

### Methylation level of the *OMT30376* gene was different between purple and red tubers

**Figure 6:**
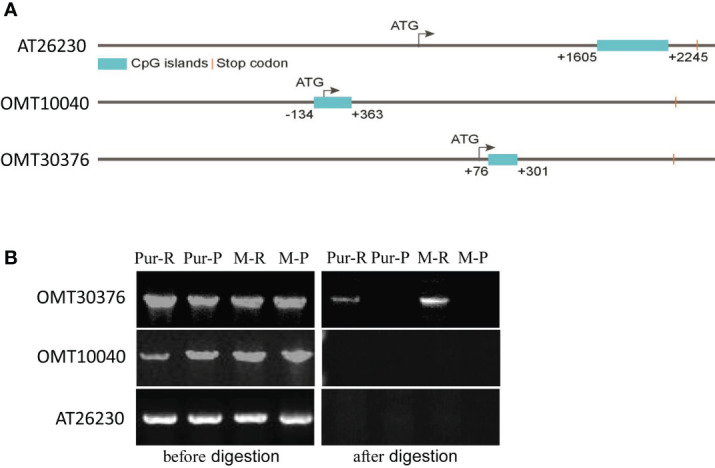
Detection of the methylation level of O-methyltransferase (OMT) and acyltransferase (AT) genes. **(A)** Distribution of CpG islands in gene sequences of *AT26230*, *OMT10040*, and *OMT30376*. **(B)** Specific primers amplified target genes in gDNA of red skin of Purple Viking tuber (Pur-R), purple skin of Purple Viking tuber (Pur-P), skin of red mutant (M-R), and skin of purple mutant (M-P) before (left) and after (right) *Hpa*II digestion.

Sequences of *OMT10040*, *OMT30376*, and *AT26230* genes and promoters were cloned from M-R and M-P. There were no significant differences in sequences of the three genes between M-R and M-P. Therefore, methylation levels of the three genes and associate 3,000-bp upstream promoter sequences were predicted. All three genes contained CpG island sequences, and the positions of the CpG island in sequences of *AT26230*, *OMT10040*, and *OMT30376* genes were 1,605–2,245, –134–363, and 76–301, respectively (**Figure 6A**), which indicated that the three genes might be methylated.

The gDNA was extracted from the red part (Pur-R) and purple part (Pur-P) of Purple Viking tuber skin and tuber skins of M-R and M-P. The gDNA of Pur-R, Pur-P, M-R, and M-P was digested by *Hpa*II, which is a restriction enzyme sensitive to methylation. Primers spanning CpG island sequences were designed, and gDNA before and after enzyme digestion was used as a template for PCR amplification to determine whether a gene sequence was methylated. Target gene fragments were amplified when gDNA before *Hpa*II digestion was used as the template. However, when digested gDNA was used as the template, the primer of the *OMT30376* gene amplified the target band in gDNA of Pur-R and M-R, but there was no target band in Pur-P and M-P (**Figure 6B**). However, target bands in *OMT10040* and *AT26230* genes were not amplified in any template after enzymatic digestion (**Figure 6B**). The results indicated that the methylation degree of the *OMT30376* gene in Pur-R and M-R was higher than that in Pur-P and M-P. Thus, the difference in degree of methylation might cause lower expression of the *OMT30376* gene in M-R than in M-P (**Figure 6B**).

### Methylation level of the *OMT30376* gene affected the transformation of anthocyanin in tubers

**Figure 7:**
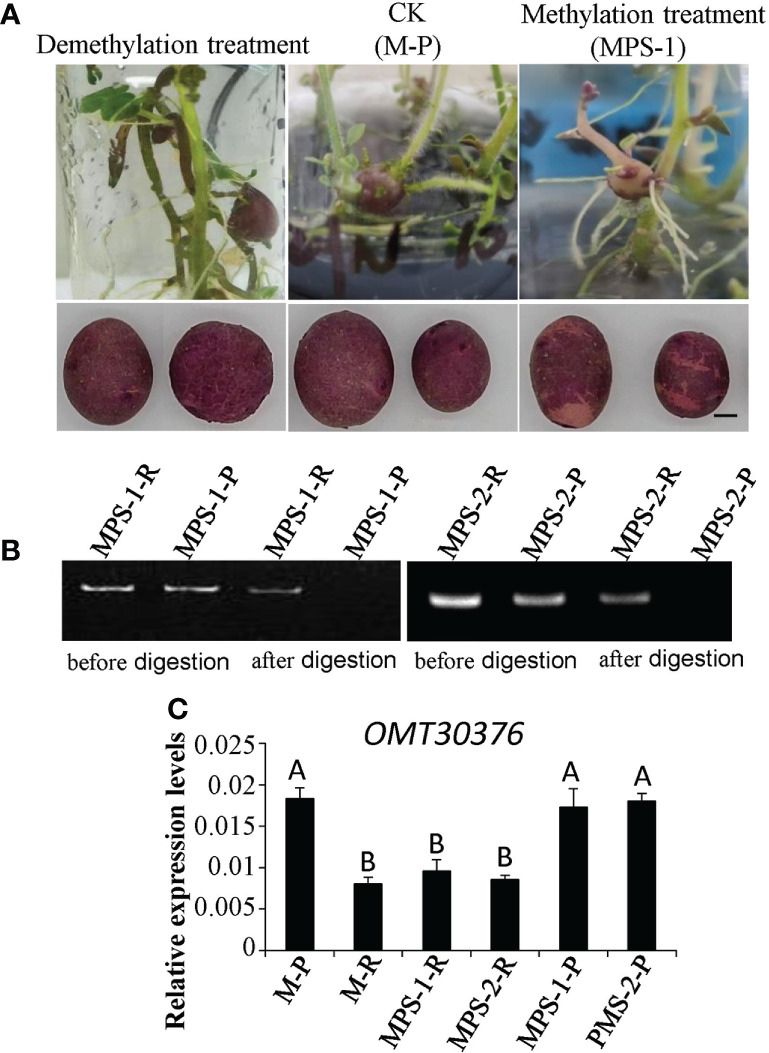
Effects of methylation treatment on transformation of anthocyanin. **(A)** Phenotypes of tubers from purple mutant (M-P) plantlets cultured on the medium containing 5-azaC (demethylation treatment), SAM (methylation treatment) (MPS), and water (CK), respectively. Bars = 1 cm. **(B)** OMT30376M-F/R primers amplified the target gene in gDNA of red skin of MPS (MPS-1-R and MPS-2-R) and purple skin of MPS (MPS-1-P and MPS-2-P) before and after *Hpa*II digestion. **(C)** Transcript levels of *OMT30376* in red and purple skin of MPS tubers. Each column represents the mean value ± standard error (*n* = 3). Different letters above bars indicate a significant difference between means (P<0.01).

The M-P tissue culture seedlings were expanded with MS medium supplemented with S-adenosylmethionine (SAM) or 5-azacytidine (5-azaC) to induce tuber formation. The phenotype of the chimeric color of red and purple (MPS) appeared in M-P potato tubers cultured in the medium containing SAM (**Figure 7A**). The three micro potatoes of MPS were planted in separately in plastic pots, and the tubers harvested also had the color skin phenotype (**Figure 7A**). However, the phenotype of the tuber cultured in the medium containing 5-azaC was purple, which was the same as that of M-P (**Figure 7A**). The gDNA of red skin (MPS-1-R and MPS-2-R) and purple skin (MPS-1-P and MPS-2-P) of MPS-1 and MPS-2 tubers was extracted, and digested with restriction enzyme *Hpa*II. The target gene was amplified with digested and undigested gDNA with *OMT30376* primers (MT30376M-F/R). The results of MPS-1 and MPS-2 were consistent and showed that the target fragment was amplified from gDNA without *Hpa*II treatment. After *Hpa*II treatment, the target fragment was amplified in MPS-1-R and MPS-2-R, but not in MPS-1-P and MPS-2-P (**Figure 7B**). Therefore, the methylation level of the *OMT30376* gene was higher in red skin of MPS than in purple skin of MPS. The expression level of *OMT30376* in MPS-1-R and MPS-2-R was similar to that in the M-R tuber, which was much lower than that in MPS-1-P, MPS-2-P and M-P (**Figure 7C**). Thus, an increase in the methylation level of the *OMT30376* gene led to a decrease in its expression, which then led to an increase in red anthocyanin accumulation.

## Discussion

Plant anthocyanin biosynthesis is regulated by many factors, such as temperature, hormonse, microRNAs, and epigenetics (Zhang et al., 2012; Yamazaki et al., 2021). To understand the regulation of potato anthocyanin biosynthesis, research has primarily focused on analysis of key gene functions and regulation mechanisms, particularly of structural genes, including *F3’5’H*, *DFR*, *ANS*, and *3GT* (Jung et al., 2005; Wei et al., 2012; Zhang et al., 2020), and MBW transcription factors (Li et al., 2014; Liu et al., 2016). However, regulation of methylation modification in potato anthocyanin biosynthesis has not been reported. In this study, transcriptome and anthocyanin metabolome analyses were performed on Purple Viking tubers with a chimeric skin phenotype and its associated red and purple-skin mutants. The metabolome analysis showed that the transformation of cyanidin and delphinidin was hindered in red tubers (**Figure 2**). Analysis of DEGs showed that expression of the *OMT30376* gene, which catalyzes the conversion of cyanidin and delphinidin, was significantly different between M-R and M-P (**Figure 5**). Further analysis showed a difference in the methylation degree of the *OMT30376* gene between M-R and M-P. In addition, M-P treated with SAM had the phenotype of the restored mutation (**Figure 7**). The results indicated that the anthocyanin difference between purple and red was caused by the methylation degree of the *OMT30376* gene. Therefore, the *OMT30376* gene is involved in the transformation of anthocyanins in potato tubers, which is consistent with the function of the grape *AOMT* gene. Transient expression of the grapevine *AOMT* gene in tobacco leaves expressing anthocyanin pigment 1 (PAP1) transcription factor from *Arabidopsis*, indicates that AOMT transforms the nonmethylated anthocyanin delphinidin 3-rutinoside induced by PAP1 into malvidin 3-rutinoside (Hugueney et al., 2009).

Epigenetic modification of anthocyanin biosynthesis regulators, especially methylation modification, is closely associated with color changes in some crops (Xu et al., 2012; Wang et al., 2013; El-Sharkawy et al., 2015). The OMT genes have key roles in methylation modification, and the multiple genes in the OMT gene family also have different effects. For example, four different OMTs isolated from petunia exhibit different kinetic properties (Jonsson et al., 1984), and of 57 candidate OMT genes identified in tomatoes, only one gene is strongly correlated with methylated anthocyanin accumulation (Roldan et al., 2014). Similar results are found in grapes. High expression of the *FAOMT* gene is detected in colored grape skin, but other OMT genes are not detected (Lucker et al., 2010). In this study, seven OMT genes were detected in the transcriptomes, and RT-qPCR results showed that only two (*OMT10040* and *OMT30376*) had lower in expression in M-R than in M-P (**Figure 5**). According to further analysis, only the difference in methylation level of the *OMT30376* gene (**Figure 6**) affected anthocyanin transformation.

The focus of most previous studies is on the effect of OMT genes on anthocyanin methylation in order to change the color performance of plants (Ageorges et al., 2006; Castellarin and Di Gaspero, 2007; Du et al., 2015). Methylation of anthocyanins was first reported in petunia at the 3′ and 5′ positions of aglycones (Jonsson et al., 1983). Subsequently, effects of OMT genes on anthocyanin methylation were determine in many species, including peony and grape (Sakata et al., 1995; Kondo et al., 2009). However, differences in regulation of different OMT genes in different phenotypes are rarely reported. In this study, the difference in methylation level of the *OMT30376* gene between M-R and M-P affected anthocyanin transformation, which is a new discovery in potato.

Methylation regulates anthocyanin synthesis primarily because of changes in methylation level of key transcription factors, which lead to changes in expression of structural genes and thus activity of those genes (El-Sharkawy et al., 2015). A decrease in the promoter methylation level of *MdMYB1*, the key transcription factor in anthocyanin biosynthesis in apples, increases the accumulation of red anthocyanin and produces a red bud sport (Xu et al., 2012). Similar results are also found in red-fleshed radish, in which methylation of the *RsMYB1* promoter leads to a decrease in gene expression and affects the synthesis and accumulation of anthocyanin (Wang et al., 2020). However, in this study, there were no significant differences in expression of key transcription factors and most key structural genes (**Figure 2**) between red and purple tubers. When methylation level and expression of the *OMT30576* gene in red and purple tubers (**Figures 5B**, **6**) and effect of methylation level on anthocyanin transformation were both considered (**Figure 7**), the difference in anthocyanin between purple and red tubers was primarily due to difference in methylation levels of the *OMT30376* gene, which affected its expression. This is the first report on how methylation of an OMT gene affects anthocyanin biosynthesis and transformation in potato tubers. In addition, the CpG of *OMT30376*, *OMT10040*, and *AT26230* are all in the coding region (**Figure 6A**), and the expression level of them in the M-R and M-P is significantly different (**Figure 5**), indicating that the difference in methylation of gene coding regions can regulate gene expression, which is consistent with the results in rice and maize (Lu et al., 2015; Tan et al., 2016). In this study, the regulation of *OMT30376* on other new structural genes and transcription factors could not be excluded, because expression of the structural gene *FLS* and the transcription factor gene *bHLH96* was significantly different between M-R and M-P (**Figure 5**). Therefore, whether *OMT30376* can regulate expression of *FLS* and *bHLH96* needs to be investigated further.

In this study, part of the MPS tuber skin was red (**Figure 7A**), and expression of the *OMT30376* gene in the red part was lower than that in the purple part (**Figure 7C**), which indicated that expression of the *OMT30376* gene was site-specific. A similar phenomenon is observed in red–purple-flowered cyclamen (Akita et al., 2011). However, there was no purple on the tuber skin of M-R plants grown on medium containing 5-azaC, indicating that there might be other regulatory mechanisms in red tubers. In addition, differences in methylation levels of *OMT10040* and *AT26230* in M-R and M-P were not detected using the current method (**Figure 6B**). The reasons for differences expression of *OMT10040* and *AT26230* in M-R and M-P need to be studied further. The function of transcription factor *bHLH96* was not clear, although its expression in M-P and M-R was significantly different (**Figure 5**). Thus, whether bHLH96 regulates anthocyanin biosynthesis and whether it regulates *OMT10040* and *AT26230* also need to be investigated further.

## Data availability statement

The data presented in the study are deposited in the Harvard Dataverse repository, and the original fpkm value of RNA-seq can be obtained through the website https://dataverse.harvard.edu/dataset.xhtml?persistentId=doi:10.7910/DVN/K24WV6.

## Author contributions

BS and HZ designed the research. YZ and XZ analyzed the data. ZZ analyzed gene expression. YZ and JL performed the methylation treatment of samples. MS found the mutant tuber. HZ and BS wrote the article. All authors contributed to the article and approved the submitted version.

## Funding

This work was supported by grants from the China Agriculture Research System of MOF and MARA (CARS-09-P07), the Natural Science Foundation of Henan (202300410152), the Training Plan for Young Backbone Teachers in the Colleges and Universities of Henan Province (2021GGJS049), and Research Funding for Young Backbone Teachers of Henan University of Science and Technology (4026-13450008).

## Conflict of interest

The authors declare that the research was conducted in the absence of any commercial or financial relationships that could be construed as a potential conflict of interest.

## Publisher’s note

All claims expressed in this article are solely those of the authors and do not necessarily represent those of their affiliated organizations, or those of the publisher, the editors and the reviewers. Any product that may be evaluated in this article, or claim that may be made by its manufacturer, is not guaranteed or endorsed by the publisher.
