# Supplementary material for: Methylation level of potato gene OMT30376 regulates tuber anthocyanin transformations
Source: Front Plant Sci. 2022 Oct 7;13:1021617. doi: 10.3389/fpls.2022.1021617 (PMC9585915; doi:10.3389/fpls.2022.1021617)
Supplement: Supplementary file 1 [file Table_1.docx]

Table S1 The anthocyanin contents in each sample

|  | M-R1 | M-R2 | M-R3 | M-P1 | M-P2 | M-P3 | PurS1 | PurS2 | PurS3 | PurF1 | PurF2 | PurF3 |
| --- | --- | --- | --- | --- | --- | --- | --- | --- | --- | --- | --- | --- |
| Rosinidin-O-hexoside | 73105 | 37989 | 52815 | 57587 | 188710 | 114300 | 90151 | 74521 | 32421 | 9 | 9 | 9 |
| Peonidin 3-O-glucoside | 684360 | 662320 | 802490 | 1079500 | 810770 | 918570 | 525770 | 781920 | 1177700 | 43912 | 54465 | 35396 |
| Delphinidin | 215030 | 90165 | 67498 | 7573 | 14245 | 14489 | 24967 | 12621 | 5034 | 25806 | 18693 | 17682 |
| Pelargonidin | 43296 | 47442 | 47922 | 10047 | 12658 | 17858 | 9025 | 8970 | 5066 | 8326 | 9734 | 6869 |
| Malvidin 3-O-glucoside | 9 | 9 | 9 | 7657400 | 2443700 | 4603900 | 1554500 | 3751100 | 8839100 | 9 | 9 | 9 |
| Petunidin 3-O-glucoside | 9 | 9 | 9 | 343130 | 645940 | 773750 | 311360 | 437090 | 470000 | 9 | 9 | 9 |
| Pelargonidin 3-O-beta-D-glucoside | 4026800 | 4608700 | 4593900 | 690580 | 343410 | 622860 | 325280 | 324970 | 372220 | 732150 | 1093800 | 300620 |
| Cyanidin | 391440 | 145560 | 220000 | 13623 | 241580 | 119310 | 363550 | 87305 | 15300 | 52373 | 67589 | 65230 |
