# Supplementary material for: Methylation level of potato gene OMT30376 regulates tuber anthocyanin transformations
Source: Front Plant Sci. 2022 Oct 7;13:1021617. doi: 10.3389/fpls.2022.1021617 (PMC9585915; doi:10.3389/fpls.2022.1021617)
Supplement: Supplementary file 2 [file Table_2.docx]

Table S2 Statistic reads of RNA-Seq data

| Sample name | Total reads | Clean reads | Total mapped(%) | Q30 (%) | GC content (%) |
| --- | --- | --- | --- | --- | --- |
| M-R1 | 42735666 | 21367833 | 89.66 | 94.61 | 43.05 |
| M-R2 | 45318136 | 22659068 | 83.84 | 94.51 | 42.65 |
| M-R3 | 42281112 | 21140556 | 80.43 | 94.89 | 42.75 |
| PurS1 | 41626540 | 20813270 | 85.14 | 94.63 | 43.17 |
| PurS2 | 40922896 | 20461448 | 86.59 | 95.17 | 42.90 |
| PurS3 | 41049898 | 20524949 | 82.06 | 94.38 | 42.44 |
| PurF1 | 48638688 | 24319344 | 88.35 | 95.25 | 43.66 |
| PurF2 | 39552366 | 19776183 | 87.90 | 94.94 | 43.28 |
| PurF3 | 54212270 | 27106135 | 88.07 | 94.97 | 43.47 |
| M-P1 | 45449192 | 22724596 | 85.35 | 94.75 | 42.67 |
| M-P2 | 42429156 | 21214578 | 80.97 | 94.59 | 42.89 |
| M-P3 | 42269982 | 21134991 | 84.73 | 95.01 | 43.09 |
